# Novel Prognostic Biomarkers in Metastatic and Locally Advanced Colorectal Cancer: Micronuclei Frequency and Telomerase Activity in Peripheral Blood Lymphocytes

**DOI:** 10.3389/fonc.2021.683605

**Published:** 2021-06-28

**Authors:** Taxiarchis Konstantinos Nikolouzakis, Elena Vakonaki, Polychronis D. Stivaktakis, Athanasios Alegakis, Aikaterini Berdiaki, Nikolaos Razos, John Souglakos, Aristidis Tsatsakis, John Tsiaoussis

**Affiliations:** ^1^ Department of Anatomy, Medical School, University of Crete, Heraklion, Greece; ^2^ Laboratory of Toxicology, Medical School, University of Crete, Heraklion, Greece; ^3^ Laboratory of Histology-Embryology, Medical School, The University of Crete, Heraklion, Greece; ^4^ Department of Medical Oncology, University General Hospital of Heraklion, and Laboratory of Translational Oncology, Medical School, University of Crete, Heraklion, Greece; ^5^ Department of Forensic Sciences and Toxicology, Medical School, University of Crete, Heraklion, Greece

**Keywords:** metastatic colorectal cancer, locally advanced rectal cancer, micronuclei frequency, telomerase activity, biomarkers, prognosis

## Abstract

**Purpose:**

Due to the current practice on colorectal cancer (CRC) management, chemoresistance is most often recognized at the end of the treatment. Therefore, effective and easy-to-use prognostic biomarkers are needed.

**Experimental Design:**

We evaluated the prognostic significance of two novel CRC biomarkers: a) micronuclei frequency (MNf) in 55 metastatic CRC (mCRC) and 21 locally advanced rectal cancer (laRC) patients using cytokinesis block micronucleus assay (CBMN assay) and b) telomerase activity (TA) in 23 mCRC and five laRC patients using TRAP-ELISA. Both biomarkers were evaluated in peripheral blood lymphocytes (PBLs) before, at the middle, and at the end of the therapy (approximately 0, 3, and 6 months) for mCRC patients before, at the end of the therapy, and after surgery for laRC patients.

**Results:**

Overall, MNf demonstrated significant prognostic value since a decrease of MNf less than 29% between middle and initial MNf measurements can discriminate between progressive and stable/responsive disease with sensitivity of 36% and specificity of 87.0% while being able to identify responsive disease with sensitivity of 72.7% and specificity of 59.3%. On the other hand, TA presented a significant trend of increase (p = 0.07) in patients with progressive disease at the middle measurement.

**Conclusions:**

The findings of this study suggest that the MN frequency may serve as a promising prognostic biomarker for the monitoring of the treatment response of patients with CRC, while TA should be evaluated in a larger group of patients to further validate its significance.

## Introduction

Colorectal cancer is considered as one of the leading causes of cancer-related morbidity and mortality worldwide for both sexes. It was estimated that in 2020 147,950 individuals would be diagnosed with CRC in the United States (70.7% would suffer from colon and 29.3% from rectal cancer) while 53,200 patients would die from the disease (1). Projection in 2021 has not improved since it is estimated that 149,500 individuals will be affected and 52,980 will die by it (2). CRC patients with distant metastases present the worst prognosis since a significant number of them develop resistance to their therapy. Unfortunately, diagnosis of chemoresistance is most often delayed, allowing for cancer progression to take place before these patients receive second or third line treatments. At the same time, healthcare systems are dealing with an immense financial burden as a result of these treatments. Therefore, it is crucial to identify accurate, cost efficient, and easy-to-use tools that will provide valuable prognostic and predictive information. A great body of evidence indicates molecular biomarkers as promising candidates for this purpose (3). In our previous work on MNf in mCRC (4), we demonstrated that in accordance to already published data (5), mCRC results in higher MNf than that of healthy individuals. Moreover, chemotherapy was found to have a direct effect on MNf as documented in the middle of the therapy. In addition, patients with complete or partial response had further decrease in MNf at the end of the treatment in contrast to those with stable or progressive disease. In fact, this finding suggests that a decrease of cancer load can be identified by the concomitant decrease of MNf, while an increase of the cancer load (due to the emergence of a chemoresistant cluster of cancer-cells) will result in an increase of MNf, but not to the same level as before treatment. Therefore, our team focused on validating the clinical and possible prognostic value of two novel biomarkers (MNf and TA) for laCRC and mCRC. These biomarkers were chosen on the basis of their close relation to chromosomal instability (CIN) and aberrant genetic function, both major hallmarks in colorectal carcinogenesis (6).

Micronuclei (MNs), also known as Howell–Jolly bodies, are particles formed during anaphase, since part of the genetic material fails to follow the rest of the chromosomes and therefore is not included in the daughter nucleus during telophase. This results in a smaller “nucleus” close to the main one. It is exhibited that MN formation can be attributed primarily to mitotic spindle failure, kinetochore damage, centromeric DNA hypomethylation, and defective control in the cell cycle system (7). Nonetheless, their presence in a healthy individual is not unusual although they tend to be more common among people adhering to unhealthy lifestyles (8). Generally, males tend to have lower MNf than females, and younger individuals have lower MNf than the older individuals (9). MNf estimation is well-established as an indicator of genetic damage of any cause, especially as an exposure biomonitoring against carcinogens (10, 11). Numerous studies have shown that MNf is a sensitive biomarker of various types of cancers such as lung, bladder, and CRC, suggesting that cancer patients exhibit higher MNf than healthy individuals (4, 5). Although a number of studies have explored MNf in CRC (12, 13), it is not clear what is the course of MNf in the long-term and especially how MNf is correlated with prognosis.

Telomerase, the regulating enzyme of telomeres’ length, is an enzymatic complex consisting of two subunits, the catalytic subunit, the human telomerase reverse transcriptase (hTERT), and a template, the telomerase RNA component (TERC). Notwithstanding that, the telomeres’ length is induced in each cell cycle in a lower rate, endorsing cellular senescence (14). In non-cancerous somatic cells, TA is undetectable or present at low levels. Cellular senescence is a key barrier against cancer, which implies that cancer cells have been transformed to immortal cells. This fact requires increased levels of TA, in order not to decrease telomere’s length. This mechanism is explained by the hTERT promoter, whose upregulated expression is promoted by differential hTERT gene expression in neoplastic and normal cells. For example, Chen et al. demonstrated that a net increase of hTERT units is possible through upregulation of SPT5, a tumor-specific hTERT promoter-binding protein encoded by the upregulated SUPT5H gene (15), while Ling Zhang et al. using the HCT-116 cell line (a KRAS mutated line), exhibited increased TA *via* upregulation of the T-STAR gene (which encodes a number of proteins responsible for multiple functions in pre-mRNA splicing, signaling, and cell cycle control) (16). Given the implication of telomeres’ length in CRC, TA has attracted scientific interest as well. Jian Zou et al. identified that telomerase is found to be activated in 90% of malignant tumors (17). Interestingly, TA has been detected in early stages of CRC which would mean that it is a determining factor during carcinogenesis (18), while increased hTERT expression and elevated plasma concentration of circulating TERT mRNA have also been identified as an unfavorable independent prognostic marker of overall survival in patients with stage II CRC (19).

## Materials and Methods

### Patients and Study Protocol

**Figure 1:**
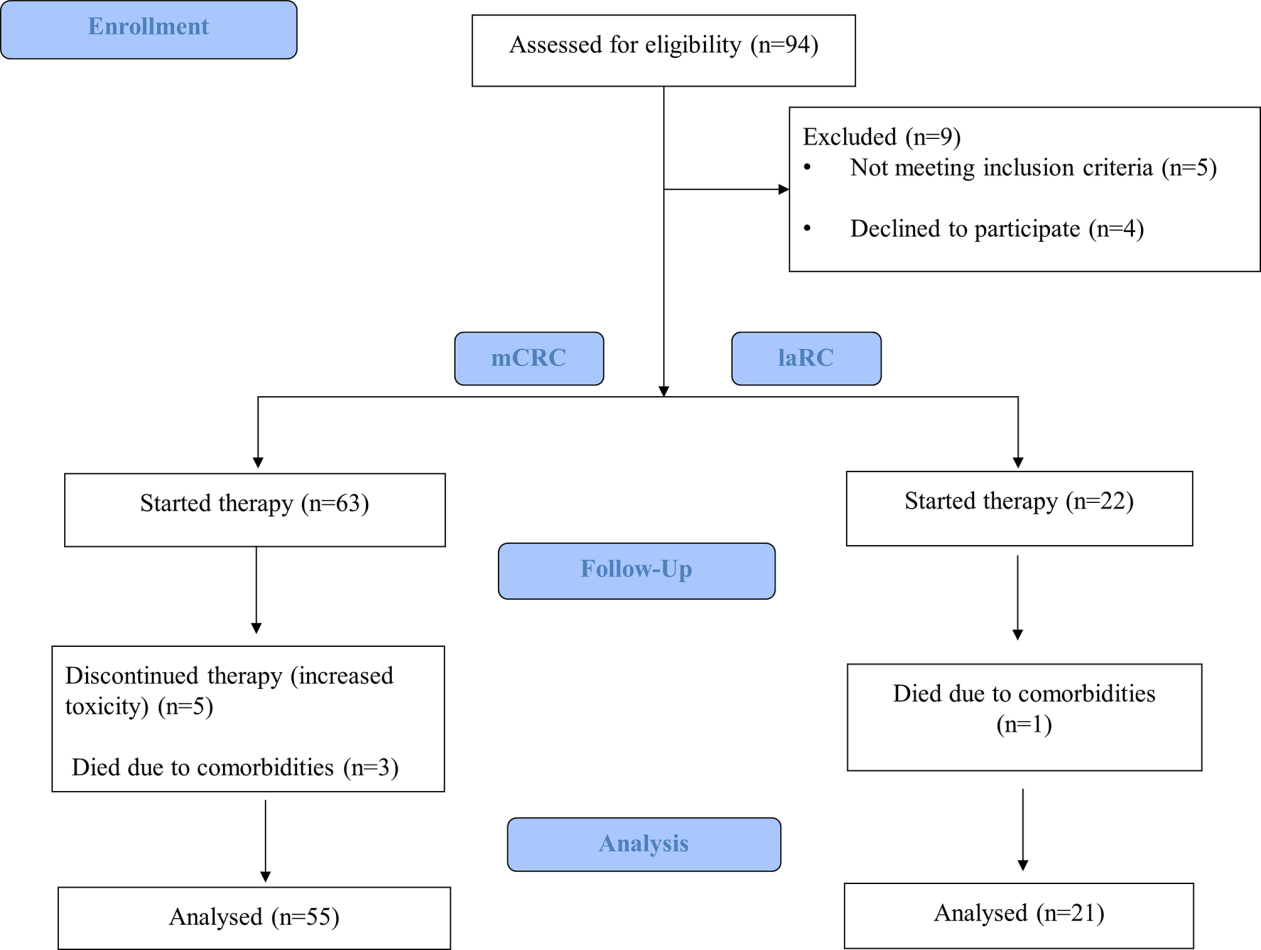
CONSORT flow diagram of our study.

From December 2016 to February 2021, 94 consecutive patients treated at the Department of Medical Oncology, University Hospital of Heraklion were evaluated for participation in this study. Inclusion criteria were: I) Patients with radiologic evidence of mCRC documented by computed tomography (CT) and/or magnetic resonance imaging (MRI) presenting measurable disease treated with first line systemic treatment according to the Hellenic Society of Medical Oncologists (HeSMO) guidelines (20), II) Patients with radiologic evidence of laRC, documented by abdominal and chest CT and abdominal and pelvic MRI presenting measurable disease and receiving induction chemotherapy according to the Hellenic Society of Medical Oncologists (HeSMO) guidelines (21). Exclusion criteria were as follows: I) Failure to complete the therapeutic regimen for any reason (toxicity, refusal of the patient, or death), II) Refusal of the patient to attend the study III) Synchronous second primary cancer at the time of enrollment and/or therapy. Out of 94 patients evaluated, 85 were found eligible and were included; however, only 76 managed to complete the study (55 mCRC and 21 laRC) due to the fact that five patients presented increased toxicity and had to stop while another four died prior to completion of the study (**Figure 1**). We decided to use the embryologic origin of each part of the colon in order to divide primary location of the lesion on the right and left sides (caecum, ascending colon, and proximal 2/3 of the transverse colon from the midgut and therefore right colon, distal third of the transverse, the descending, sigmoid colon, and the rectum from the hindgut and therefore left colon). The protocol for this study has been approved by the Ethics Committee for Patients and Biological Material of the University Hospital of Heraklion (Heraklion, Greece). All participants signed an informed consent agreement. All samples generated by this study were anonymized, and personal data was managed according to the EU General Data Protection Regulation (GDPR).

### Therapy Selection for mCRC

Based on the chemotherapeutic protocol that was selected, mCRC patients received one of the following therapies: I) folinic acid with 5-fluorouracil and oxaliplatin (FOLFOX), II) folinic acid with 5-fluorouracil and irinotecan (FOLFIRI), or III) folinic acid with 5-fluorouracil, oxaliplatin, and irinotecan (FOLFOXIRI). Based on the genetic profile of each patient, a biological factor could be used.

### Therapy Selection for laRC

Patients with laRC were informed about their therapeutic strategy: Induction chemotherapy and then operation for RC. Chemotherapeutic protocols were FOLFOX and capecitabine and oxaliplatin (CAPOX).

### Response Evaluation

The RECIST criteria version 1.1 were used as the gold standard for the evaluation of the treatment response (22). Using these criteria, patients were evaluated at the end of the therapy and were divided into four subgroups: complete response (disappearance of all target lesions), partial response (at least a 30% decrease in the sum of diameters of target lesions, taking as reference the baseline sum diameters), stable disease (neither sufficient shrinkage to qualify for partial response nor sufficient increase to qualify for partial disease, taking as reference the smallest sum diameters) and progression (at least a 20% increase in the sum of diameters of target lesions, taking as reference the smallest sum on study or the appearance of one or more new lesions).

### Blood Sampling

Peripheral blood samples were collected at predetermined time-points. For mCRC patients, these were before the beginning of the treatment, in the middle of it, and at the end of treatment (approximately 0, 3, and 6 months of treatment, respectively). For laRC patients, samples were taken before the beginning of chemotherapy and at the end. We also took blood samples two weeks postoperatively from 12 laRC patients. All blood samples were stored in 5°C until processing within 48 h from sampling.

### Control Group

The control group was constituted by 25 healthy individuals with no medical condition after having normal colonoscopy. All individuals provided an informed written consent. Inclusion criteria were: age between 45 and 75 years old, medical history free of cancer, autoimmune diseases, diabetes mellitus type I or II, and chronic obstructive pulmonary disease (COPD), non-smokers or no smoking habits for the last ten years, and no consumption of immune-modifying medication. Exclusion criteria were the presence of the above-mentioned diseases, direct exposure at any time (domestic or occupational) to pesticides, herbicides, organic solvents, or any persistent organic pollutant; for women, additional exclusion criteria were the use of oral contraceptives and the will not to participate in the study.

### MN Test

Laboratory preparation for MNf quantification follows the description we have presented earlier (4). Standard criteria were used for scoring the BNMN: 1) the cells should be binucleated, 2) the two nuclei in a binucleated cell should have intact nuclear membranes and be situated within the same cytoplasmic boundary, 3) the two nuclei in a binucleated cell should be approximately equal in size, staining pattern, and staining intensity, 4) the two nuclei within a BN cell may be attached by a fine nucleoplasmic bridge which is no wider than one-fourth of the largest nuclear diameter, 5) the two main nuclei in a BN cell may touch but ideally should not overlap each other. A cell with two overlapping nuclei can be scored only if the nuclear boundaries of each nucleus are distinguishable, 6) the cytoplasmic boundary or membrane of a binucleated cell should be intact and clearly distinguishable from the cytoplasmic boundary of adjacent cells. One thousand binucleated (BN) cells with an intact cytoplasm were scored per slide for each sample in order to calculate the number of binucleated cells with micronuclei (BNMN) and thereafter MNf. Criteria for scoring MN were: 1) the diameter of MN in human lymphocytes usually varies between 1/16 and 1/3 of the mean diameter of the main nuclei which corresponds to 1/256 and 1/9 of the area of one of the main nuclei in a BN cell, respectively, 2) MNs are round or oval in shape, 3) MNs are non-refractile, and they can therefore be readily distinguished from artefacts such as staining particles, 4) MNs are not linked or connected to the main nuclei, 5) MNs may touch but not overlap the main nuclei, and the micronuclear boundary should be distinguishable from the nuclear boundary, 6) MNs usually have the same staining intensity as the main nuclei but occasionally staining may be more intense (23, 24). The cytokinesis block proliferation index (CBPI) is given by the following equation:

$$CBPI=\frac{M_{1}+2M_{2}+3(M_{3}+M_{4})}{N}$$

where M1, M2, M3, and M4 correspond to the number of cells with one, two, three, and four nuclei, respectively, and ‘N’ is the total number of cells. For CBPI calculation, 2,000 cells were counted. CBPI is a tool that is used in order to better understand BNMN results from cell cultures where cytochalasin B is used. Moreover, it is able to provide substantial information regarding possible cytotoxic effects (necrosis, apoptosis, or cytostasis) on the cell culture induced by any chemical agents. If CBPI remains close to the numeric value of one, then there is no cytotoxic event. Moreover, should it remain almost the same between time-points, then MNf results are comparable, and any fluctuation of MNf can be attributed solely to the parameter of interest (in our case CRC and/or the systemic treatment). These parameters were calculated, in order to determine the possible cytotoxic effects, as previously described (25–27).

### Telomerase Activity Estimation: Polymerase Chain Reaction–Enzyme Linked Immune Sorbent Assay

TA was measured in 28 patients, whose characteristics are presented in **Table 1** at three time points: before (at the beginning of the therapy), middle (at the middle of therapy for mCRC, and at the end of therapy for laRC) and after (at the end of therapy for mCRC and after surgery for laRC). Telomerase activity was evaluated by photometric enzyme immunoassay for the detection of telomerase activity, utilizing the Telomeric Repeat Amplification Protocol (TRAP). Peripheral blood mononuclear cells (PBMCs) were harvested from the blood samples by Ficoll–Hypaque gradient centrifugation as described by Tsirpanlis et al. (28), and TRAP-ELISA was conducted using TeloTAGGG Telomerase PCR ELISA (Roche Diagnostics Corp., Indianapolis, IN, USA) following the manufacturer’s manual (29) and Kara et al. (30). In order to achieve higher data reliability, all samples were tested in triplicates. TA was expressed as a totality and as per outcome (progression, stable, partial and complete response).

**Table 1:** Patient characteristics for MNf and TA groups are presented [sex, age, ECOG performance status, chemotherapy, biologic agent, disease response based on the RECIST criteria, KRAS status, NRAS status, BRAF status, mismatch repair (MMR) status, location of the primary lesion, number of metastatic sites].

		MNf		p	TA		P
		mCRC	laRC		mCRC	laRC	
**Total Number of patient**		55	21		23	5	
**Sex,** n (%)	**Males**	28 (50.9)	16 (76.2)	0.068	9 (39.0)	3 (60.0)	0.393
	**Females**	27 (49.1)	5 (23.8)		14 (61.0)	2 (40.0)	
**Age,** n (%)	**≤40**	0 (0.0)	1 (4.8)	0.435	1 (4.8)	1 (20.0)	0.173
	**41-55**	13 (23.6)	5 (23.8)		5 (23.8)	3 (60.0)	
	**56-70**	24 (43.6)	8 (38.1)		8 (38.1)	1 (20.0)	
	**70+**	18 (32.7)	7 (33.3)		7 (33.3)	0 (0.0)	
**ECOG performance** **status,** n (%)	**0**	22 (95.6)	47 (85.5)	0.199	19 (86.4)	5 (100.0)	0.08
	**1**	1 (4.3)	8 (14.5)		3 (13.6)	0 (0.0)	
**Chemotherapy,** n(%)	**FOLFOX**	29 (52.7)	12 (57.0)	0.98*	10 (43.5)	4 (80.0)	0.14*
	**FOLFIRI**	22 (40.0)	0 (0.0)		13 (36.3)	0 (0.0)	
	**FOLFOXIRI**	4 (7.3)	0 (0.0)		0 (0.0)	0 (0.0)	
	**CAPOX**	0 (0.0)	9 (43.0)		0 (0.0)	1 (20.0)	
**Biological agent,** n (%)	**Bevacizumab**	19 (34.5)		NA	7 (30.0)		NA
	**Aflibercept**	5 (9.0)			5 (22.0)		
	**Cetuximab**	9 (16.5)			6 (26.0)		
	**Panitumumab**	7 (12.7)			0 (0.0)		
	**No agent**	15 (27.2)			5 (22.0)		
**Disease response,** n (%)	**Progression**	13 (23.6)	2 (9.8)	0.08	6 (20.0)	0 (0.0)	0.25
	**Stable disease**	18 (32.7)	5 (23.8)		7 (30.0)	3 (60.0)	
	**Partial response**	23 (41.8)	11 (52.4)		9 (40.0)	1 (20.0)	
	**Complete response**	1 (1.8)	3 (14.3)		1 (4.0)	1 (20.0)	
**KRAS,** n (%)	**WT**	25 (45.5)	9 (39.1)	0.61**	Unknown		
	**exon 2 mut**	18 (32.7)	6 (26.1)		***		
	**exon 3 mut**	0 (0.0)	0 (0.0)				
	**exon 4 mut**	1 (1.8)	1 (4.3)				
	**Unknown**	11 (20.0)	7 (30.4)				
**NRAS,** n (%)	**WT**	42 (76.4)	15 (65.2)	0.31**	Unknown		
	**Mutation**	2 (3.6)	2 (8.7)				
	**Unknown**	11 (20.0)	6 (26.1)				
**BRAF,** n (%)	**WT**	41 (74.5)	15 (65.2)	0.48**	Unknown		
	**V600E mut**	4 (7.3)	1 (4.3)				
	**Unknown**	11 (20.0)	7 (30.4)				
**Mismatch repair** **status,** n (%)	**Proficient**	12 (58.2)	21 (91.3)	0.01	Unknown		
	**Deficient**	2 (3.6)	1 (4.3)				
	**Unknown**	21 (38.2)	1 (4.2)				
**Location of** **primary lesion,** n (%)	**Left**	42 (76.4)	19 (83.0)	0.54	21 (100)	5 (100)	NA
	**Right**	13 (23.6)	4 (17.0)		0 (0.0)	0 (0.0)	
**Metastatic sites** [median/mean (range)]	**Liver**	3.6/4.4 (0–20)		NA			NA***
	**Lung**	3.2/3.5 (0–11)					
	**Lymph nodes**	0/3.2 (0–14)			0/3.2 (0–6)		
	**Peritoneum**	0/3.6 (0–8)					

*p-values from comparison of FOLFOX with the rest of chemotherapies, *p-values from comparison between wild-type and mutations,***Unknown: There were no data , ****NA, not applicable.

### Statistical Analysis

Frequency data were analyzed using non-parametric statistics. Pearson’s Chi-square test (χ^2^) was applied to estimate differences in proportions of patients’ and disease characteristics (**Table 1**). In order to examine TA differences and percentage differences of MNf (%DMNf) between two groups (*e.g.* mCRC *vs* IaRC), the Mann–Witney test was applied. Whereas, in order to examine TA differences and %DMNf for more than two groups (*e.g.* disease response) Kruskal–Wallis comparisons were applied. Comparison of counts of MNf was assessed using G-test when bivariate comparisons of before, middle, and after therapy sampling points were made. The Chi-squared test was used for the analysis of the CBPI values., Due to the small number of cases, a crude discrimination limit between responses was established using %DMNf as an indicator. The %DMNf definition between middle and before was set by the formula

$$\%DMNf=\frac{MNf_{middle}-MNf_{before}}{MNf_{before}}100\%$$

ROC curve analysis, corresponding diagrams of sensitivity *vs* 1-specificity were applied between %DMNf in a binary response (progressive *vs* stable/partial/complete response and progressive/stable *vs* partial/complete response) according to %DMNf.

IBM SPSS Statistics 26.0 and OpenEpi 3.01 open source epidemiological program (https://www.openepi.com/Menu/OE_Menu.htm) were used for statistical analysis of data and sensitivity analysis. A level of 0.05 was set as level of acceptance.

## Results

As presented in **Table 1**, we prospectively studied 76 CRC patients, 55 diagnosed with mCRC and 21 with laRC. In our mCRC group, 29 patients were treated with FOLFOX, 22 with FOLFIRI, and four with FOLFOXIRI (52.7, 40, and 7.3% respectively), while 40 patients received an additional treatment with a biological agent (cetuximab, aflibercept, bevacizumab or panitumumab) based on their genetic profile; 19 were treated with bevacizumab, five with aflibercept, nine with cetuximab, and seven with panitumumab (34.5, 9, 16.3, and 12.7% accordingly). On the other hand, in our laRC group, 12 were treated with FOLFOX (57%) and nine with CAPOX (43%). Based on RECIST criteria for disease response evaluation, our data suggest that the mCRC group had the following results: 13 patients exhibited progressive disease, 18 had stable disease, 23 had partial response, while one had complete response (23.6, 32.7, 41.8, and 1.8% respectively). Regarding the laRC group, two patients had progressive disease, five were stable, 11 presented partial response, and three had complete response (9.5, 23.8, 52.4, and 14.3% respectively). Finally, 32 of the mCRC patients had left-sided primary lesion (76.4%) while 13 had right sided primary lesion (23.63%). We further conducted comparative analysis between mCRC and laRC groups for those characteristics where no data were missing. All comparisons showed that there is an adjustment in demographics and patient’s data with the exception of mismatch repair status in MNf dataset (p=0.01).

### MN Frequency Evaluation

**Figure 2:**
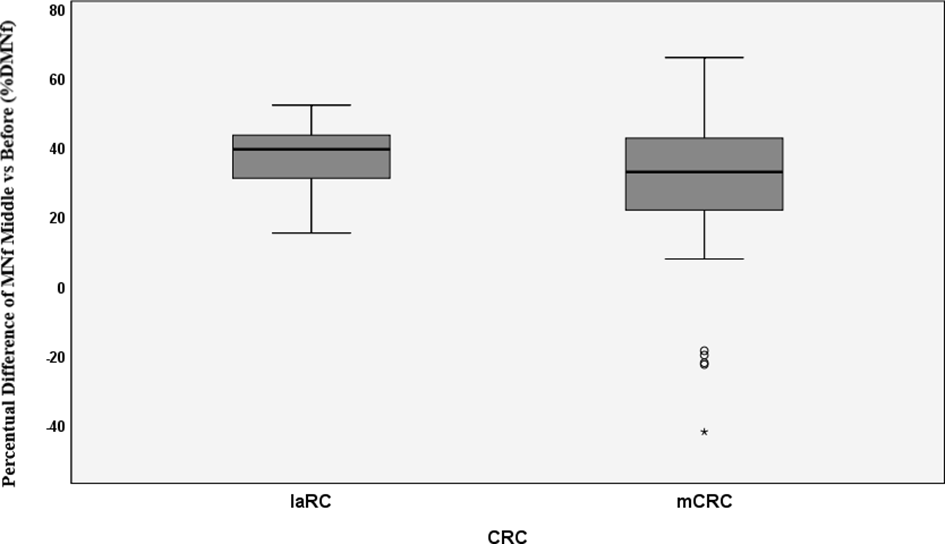
Percentual difference between middle and before measurements of MNf for metastatic colorectal cancer (mCRC) and locally advanced rectal cancer (laRC). * = extreme value as marked in SPSS.

MNf was measured for all patients in our study, and their characteristics are presented in **Table 1**. Our data from the MN assay on control group, mCRC, and laRC patients are presented in **Tables 2**–**4** respectively. Based on the absence of a significant difference of the CBPI values between: A) CRC groups and control and B) each sampling point for each response group, we are able to assume that our data is the result of the cancer itself and the different treatments. Our data indicates that MNf is significantly higher in patients with mCRC or laRC than in healthy individuals (G = 41.1, p < 0.0001 and G = 33.76, p < 0.0001 respectively). Moreover, there was no significant difference between mCRC and laRC groups, especially regarding before and middle sampling points (**Figure 2**). MNf analysis for the whole mCRC group revealed that a borderline significance is extracted when the middle of the treatment (middle) is compared to the beginning of the treatment (ZFORE) (p = 0.05), while MNf did not exhibit a further significant decrease at the end point (after) (**Table 3**). After stratification of patients according to their disease response, a relative pattern of a steady drop of MNf was observed. In detail, even though patients with progressive and stable disease exhibit an insignificant decrease of their MNf in the middle (G = 1.60, p = 0.200 and G = 3.48, p = 0.060 respectively) and at the end of their treatment (G = 2.13, p = 0.14 and G = 3.55, p = 0.06 respectively), those with disease progression had lower decrease of MNf than those with stable disease (**Table 3**). On the contrary, patients with partial response presented a statistically significant decrease of their MNf both at the middle and at the end of the treatment (G = 5.16, p = 0.02 and G = 3.94, p = 0.04 respectively) (**Table 3**). However, since there was only one patient with complete response, no statistical analysis of his data was done even though his MNf grossly followed the decreasing trend of those with partial response (before the treatment MNf was 28; at the middle, 18; and at the end, 15). Regarding the laRC group, since our primary objective was to evaluate our biomarkers’ prognostic value of therapy response, we tested for MNf after laRC surgery in approximately half of our patients (12 out of 21). Therefore, a difference in counted BN cells is observed for the sampling point “surgery” relative to the other two sampling points (**Table 4**). However, our data indicates a similar decreasing trend as observed in the mCRC group. In detail, a significant decrease of MNf (G = 4.01, p = 0.04) is found at the end of the treatment (sampling point “after”) in relation to the beginning (**Table 4**). However, even though patients maintain lower MNf than what they had before treatment, a slight increase is observed after surgery (G = 3.00, p = 0.08) (**Table 4**). When divided in subgroups, our data indicates a steady decrease of MNf that positively correlates to the disease response. Patients with progressive disease present a lower decrease of MNf after treatment (p = 0.24) than those patients with stable disease (G = 2.82, p = 0.09), partial response (G = 4.50, p = 0.03) or complete response (G = 4.77 p = 0.02) (**Table 4**).

**Table 2:** Statistical analysis of the mean BNMN and MNf of healthy controls and (A) mCRC/laRC patients at the beginning (before).

	Groups	BN cells scored	Mean ± SE	G	P
**BNMN**	**Control**	25,000	7.28 ± 1.06		
	**mCRC Before**	55,000	29.3 ± 9.07	38.00	**<0.001**
	**laRC Before**	21,000	26.4 ± 4.82	30.15	**<0.001**
**MN cells**	**Control**	25,000	8.18 ± 1.11		
	**mCRC Before**	55,000	32.3 ± 9.97	41,1	**<0.001**
	**laRC Before**	21,000	29.6 ± 5.21	33,76	**<0.001**
**CBPI ± SE** *(Mean ± SE)* ** **	**Control**	1.29 ± 0.03			
	**mCRC Before**	1.32 ± 0.004			
	**laRC Before**	1.36 ± 0.007			

Values in bold font indicate statistically significant differences (P < 0.05) compared with the controls. G indicates 2POi ln(Oi/Ei), where ‘Oi’ is the observed frequency in a cell, ‘Ei’ is the expected frequency under the null hypothesis, ‘ln’ denotes the natural logarithm and the sum is taken over all non-empty cells. SE, standard error; BN, binucleated cells (for each patient 1,000 BN cells were scored); BNMN, binucleated cells with micronuclei.

**Table 3:** Statistical analysis of the mean BNMN and MNf of mCRC patients at the beginning (before), the middle (inter) and at the end (after) of the treatment for all mCRC patients and according to their disease response (progressive disease, stable disease, partial response, and complete response).

mCRC		Sampling Point	BN cells scored	Mean ± SE	G	P
**All patients**	**BNMN**	**Before**	55,000	29.3 ± 9.07		
		**Inter**	55,000	19.4 ± 6.86	3.90	**0.04**
		**After**	55,000	19.6 ± 8.46	3.73	0.05
	**MN cells**	**Before**	55,000	32.3 ± 9.97		
		**Inter**	55,000	22.2 ± 8.28	3.65	0.05
		**After**	55,000	22.6 ± 9.72	3.35	0.06
	**CBPI ± SE** *(mean ± SE)*	**Before**	1.32 ± 0.004			
		**Inter**	1.32 ± 0.004			
		**After**	1.30 ± 0.002			
**Progressive disease**	**BNMN**	**Before**	13,000	28.8 ± 10.03		
		**Inter**	13,000	21.3 ± 6.3	2.20	0.14
		**After**	13,000	20.9 ± 9.66	2.46	0.11
	**MN cells**	**Before**	13,000	31.3 ± 10.76		
		**Inter**	13,000	24.6 ± 10.93	1.60	0.20
		**After**	13,000	23.6 ± 10.92	2.13	0.14
	**CBPI ± SE** *(mean ± SE)*	**Before**	1.29 ± 0.001			
		**Inter**	1.32 ± 0.004			
		**After**	1.31 ± 0.003			
**Stable disease**	**BNMN**	**Before**	18,000	30,2 ± 8,38		
		**Inter**	18,000	20,3 ± 6,4	3.77	0.05
		**After**	18,000	20.1 ± 8.77	3.93	**0.04**
	**MN cells**	**Before**	18,000	33.1 ± 9.35		
		**Inter**	18,000	23.1 ± 7.39	3.48	0.06
		**After**	18,000	23 ± 10.02	3.55	0.06
	**CBPI ± SE** *(mean ± SE)*	**Before**	1.32 ± 0.003			
		**Inter**	1.31 ± 0.002			
		**After**	1.30 ± 0.001			
**Partial Response**	**BNMN**	**Before**	23,000	29.0 ± 9.54		
		**Inter**	23,000	17.8 ± 5.98	5.15	**0.02**
		**After**	23,000	18.7 ± 7.88	4.29	**0.03**
	**MN cells**	**Before**	23,000	32,5 ± 10.51		
		**Inter**	23,000	20.6 ± 7.4	5.16	**0.02**
		**After**	23,000	22.0 ± 9.30	3.94	**0.04**
	**CBPI ± SE** *(mean ± SE)*	**Before**	1.33 ± 0.006			
		**Inter**	1.31 ± 0.005			
		**After**	1.30 ± 0.001			

Values in bold font indicate statistically significant differences (P < 0.05) compared with the controls or as indicated. G indicates 2POi ln(Oi/Ei), where ‘Oi’ is the observed frequency in a cell, ‘Ei’ is the expected frequency under the null hypothesis, ‘ln’ denotes the natural logarithm and the sum is taken over all non-empty cells. SE, standard error; BN, binucleated cells (for each patient 1,000 BN cells were scored); BNMN, binucleated cells with micronuclei.

**Table 4:** Statistical analysis of the mean BNMN and MNf of laRC patients at the beginning (before), the end of the treatment (after) and after surgery (surgery) for all laRC patients and according to their disease response (progressive disease, stable disease, partial response and complete response).

laRC		Sampling Point	BN cells scored	Mean ± SE	G	P
**All patients**	**BNMN**	**Before**	21,000	26.4 ± 4.82		
		**After**	21,000	16.9 ± 4.00	4,01	**0.04**
		**Surgery**	12,000	17.4 ± 3.57	3.57	0.06
	**MN cells**	**Before**	21,000	29.6 ± 5.2		
		**After**	21,000	19.7 ± 4.7	3.85	**0.04**
		**Surgery**	12,000	20.8 ± 5.48	3.00	0.08
	**CBPI ± SE** *(mean ± SE)*	**Before**	1.36 ± 0.007			
		**After**	1.32 ± 0.003			
		**Surgery**	1.27 ± 0.003			
**Progressive disease**	**BNMN**	**Before**	2,000	27.5 ± 6.36		
		**After**	2,000	21.5 ± 4.95	1.45	0.22
		**Surgery**	1,000	16.0 ± 5.1	5.80	**0.01**
	**MN cells**	**Before**	2,000	31.3 ± 10.76		
		**After**	2,000	23.5 ± 6.36	1.35	0.24
		**Surgery**	1,000	17.0 ± 5.2	6.42	**0.01**
	**CBPI ± SE** *(mean ± SE)*	**Before**	1.30 ± 0.001			
		**After**	1.40 ± 0.005			
		**Surgery**	1.26 ± 0.002			
**Stable disease**	**BNMN**	**Before**	5,000	28.7 ± 4.72		
		**After**	5,000	19.7 ± 3.25	3.25	0.07
		**Surgery**	3,000	24.0 ± 4.24	0.83	0.36
	**MN cells**	**Before**	5,000	32.7 ± 4.62		
		**After**	5,000	23.7 ± 4.72	2.82	0.09
		**Surgery**	3,000	27.0 ± 2.83	1.09	0.29
	**CBPI ± SE** *(mean ± SE)*	**Before**	1.30 ± 0.001			
		**After**	1.40 ± 0.005			
		**Surgery**	1.26 ± 0.002			
**Partial Response**	**BNMN**	**Before**	11,000	24.8 ± 4.14		
		**After**	11,000	14.9 ± 2.93	4.71	**0.02**
		**Surgery**	6,000	14.4 ± 2.64	5.25	**0.02**
	**MN cells**	**Before**	11,000	27.8 ± 4.30		
		**After**	11,000	17.5 ± 3.18	4.50	**0.03**
		**Surgery**	6,000	17.9 ± 3.18	4.14	**0.04**
	**CBPI ± SE** *(mean ± SE)*	**Before**	1.35 ± 0.008			
		**After**	1.30 ± 0.001			
		**Surgery**	1.30 ± 0.010			
**Complete response**	**BNMN**	**Before**	3,000	30.0 ± 6.25		
		**After**	3,000	18.7 ± 5.50	5.05	**0.02**
		**Surgery**	2,000	22.0 ± 4.24	2.41	0.11
	**MN cells**	**Before**	3,000	33.7 ± 7.37		
		**After**	3,000	22.0 ± 6.08	4.77	**0.02**
		**Surgery**	2,000	22.0 ± 4.24	4.77	**0.02**
	**CBPI ± SE** *(mean ± SE)*	**Before**	1.45 ± 0.001			
		**After**	1.32 ± 0.001			
		**Surgery**	1.22 ± 0.001			

Values in bold font indicate statistically significant differences (P < 0.05) compared with the controls or as indicated. G indicates 2POi ln(Oi/Ei), where ‘Oi’ is the observed frequency in a cell, ‘Ei’ is the expected frequency under the null hypothesis, ‘ln’ denotes the natural logarithm and the sum is taken over all non-empty cells. SE, standard error; BN, binucleated cells (for each patient 1,000 BN cells were scored); BNMN, binucleated cells with micronuclei.

### Evaluation of MNf as a Prognostic Biomarker

The prognostic significance of MNf in mCRC and laRC patients was roughly established using ROC curve analysis. Variation of MNf expressed as %DMNf between initial and middle measurements was estimated by setting binary outcome variables for two scenarios: Progressive disease *vs* stable/partial/complete response (scenario 1) and progressive/stable disease *vs* partial/complete response (scenario 2). For scenario 1, the best set of sensitivity and specificity was found at 29% difference between middle and initial MNf measurements (sensitivity 36% and specificity 87.0%), while the highest specificity (87.2%) was achieved at 31% reduction of MNf. For scenario 2, the best set of sensitivity and specificity was found again at 29% difference between middle and initial MNf measurements (sensitivity 72.7% and specificity 59.3%), and the highest specificity (59.6%) was found for 31% reduction of MNf (**Table 5**).

**Table 5:** Sensitivity and specificity values for three different cutoff points of percentage difference of MNf.

Limit	Outcome							
	Prog. (N_T_ = 15)		S.D./P.R./C.R. (N_T_ = 61)		Prog./S.D. (N_T_ = 38)		P.R./C.R. (N_T_ = 38)	
	N	%	N	%	N	%	N	%
**≤33%**	9	25.0%	27	75.0%	20	55.6%	16	44.4%
**>33%**	6	15.0%	34	85.0%	18	45.0%	22	55.0%
**≤31%**	9	31.0%	20	69.0%	19	65.5%	10	34.5%
**>31%**	6	12.8%	41	87.2%	19	40.4%	28	59.6%
**≤29%**	8	36.4%	14	63.6%	16	72.7%	6	27.3%
**>29%**	7	13.0%	47	87.0%	22	40.7%	32	59.3%

It can be seen that when the outcome is progressive disease vs stable/ partial/complete response, the best set of sensitivity–specificity is found at 29% difference between middle and initial MNf measurements (sensitivity 36.4% and specificity 87.0%). The highest specificity (87.2%) was found for 31% reduction of MNf. When the outcome was set between stable/progressive disease vs partial/complete response, the best set of sensitivity and specificity variables was found for 29% difference (sensitivity 72.7% and specificity 59.3%). NT, total number of patients; Prog., progression; S.D., stable disease; P.R., partial response; C.R., complete response.

### Telomerase Activity

As presented in **Table 6**, based on the non-parametric analysis (Kruskal–Wallis), there is no significant difference between patients’ mean TA before the beginning of their therapy (p = 0.256) [progressive disease: 2.1 ± 1.6 (95% CI: 0.4–3.8), stable disease: 1.4 ± 1,7 (95% CI: 0.2–2.6), partial response: 0.8 ± 1.0 (95% CI: 0.1–1.5), complete response: 0.6 ± 0.6 (95% CI: −5.2 to 6,4)], at the middle sampling point (p = 0.072) [progressive disease: 2.8 ± 0.8 (95%CI: 1.9–3.7), stable disease: 1.5 ± 1.3 (95% CI: 0.5–2.4), partial response: 1.1 ± 1.4 (95% CI: 0.1–2.1), complete response: 0.2 ± 0.1 (95% CI: −1.0 to 1.4)] or at the third sampling point (p = 0.096) [progressive disease: 2.5 ± 1.3 (95% CI: 1.1–3.9), stable disease: 1.1 ± 1.2 (95% CI:0.2–2.0), partial response: 1.1 ± 1.3 (95% CI: 0.2–2.0), complete response: 0.9 ± 1.2 (95% CI: −0.2 to 12.0)]. However, as presented in **Table 6** the mean of patients who eventually developed progressive disease, exhibited an overall higher level of TA in relation to all other response groups before the beginning of the therapy. Thereafter, TA was increased and remained elevated during the middle and third sampling points respectively.

**Table 6:** Telomerase activity measured at three time points, before (before the initiation of the therapy), middle (at the middle of therapy for mCRC and at the end of therapy for laRC), and after (at the end of the therapy for mCRC and after surgery for laRC) is presented for all CRC cases.

Telomerase Activity		n	Mean	SD	95%		Minimum	Maximum	p
					LL	UL			
**Before**	**Progression**	6	2.1	1.6	0.4	3.8	0.1	3.7	0.256
	**Stable disease**	10	1.4	1.7	0.2	2.6	0.0	3.8	
	**Partial response**	10	0.8	1.0	0.1	1.5	0.0	2.4	
	**Complete Response**	2	0.6	0.6	−5.2	6.4	0.2	1.1	
	**Total**	28	1.3	1.4	0.7	1.8	0.0	3.8	
**Middle**	**Progression**	6	2.8	0.8	1.9	3.7	1.7	3.7	0.072
	**Stable disease**	10	1.5	1.3	0.5	2.4	0.0	3.5	
	**Partial response**	10	1.1	1.4	0.1	2.1	0.0	3.2	
	**Complete Response**	2	0.2	0.1	−1.0	1.4	0.1	0.3	
	**Total**	28	1.5	1.4	1.0	2.1	0.0	3.7	
**After**	**Progression**	6	2.5	1.3	1.1	3.9	0.1	3.6	0.096
	**Stable disease**	10	1.1	1.2	0.2	2.0	0.0	3.3	
	**Partial response**	10	1.1	1.3	0.2	2.0	0.0	3.3	
	**Complete Response**	2	0.9	1.2	−10.2	12.0	0.0	1.8	
	**Total**	28	1.4	1.3	0.9	1.9	0.0	3.6	

p-values were estimated with Kruskal–Wallis test. n, number of patients.

## Discussion

**Figure 3:**
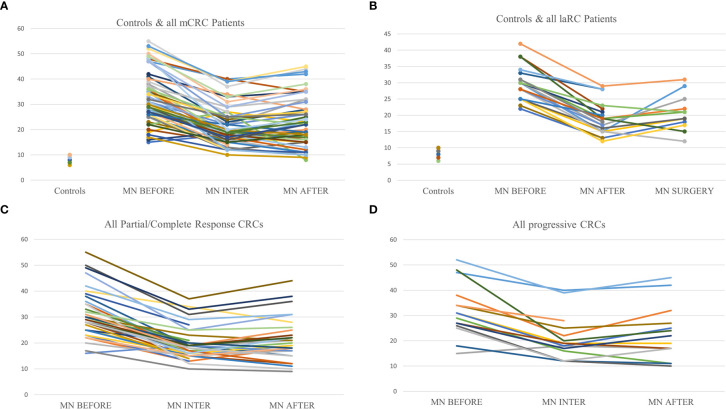
Graphic representation of MNf in controls and the trends between sampling points for every patient. **(A)** Presentation of MNf trend for controls and all mCRC patients; **(B)** presentation of MNf trend for controls and laRC patients; **(C)** presentation of MNf trend for all patients (mCRC and laRC) with partial or complete response; **(D)** presentation of MNf trend for all patients (mCRC and laRC) with progressive disease.

**Figure 4:**
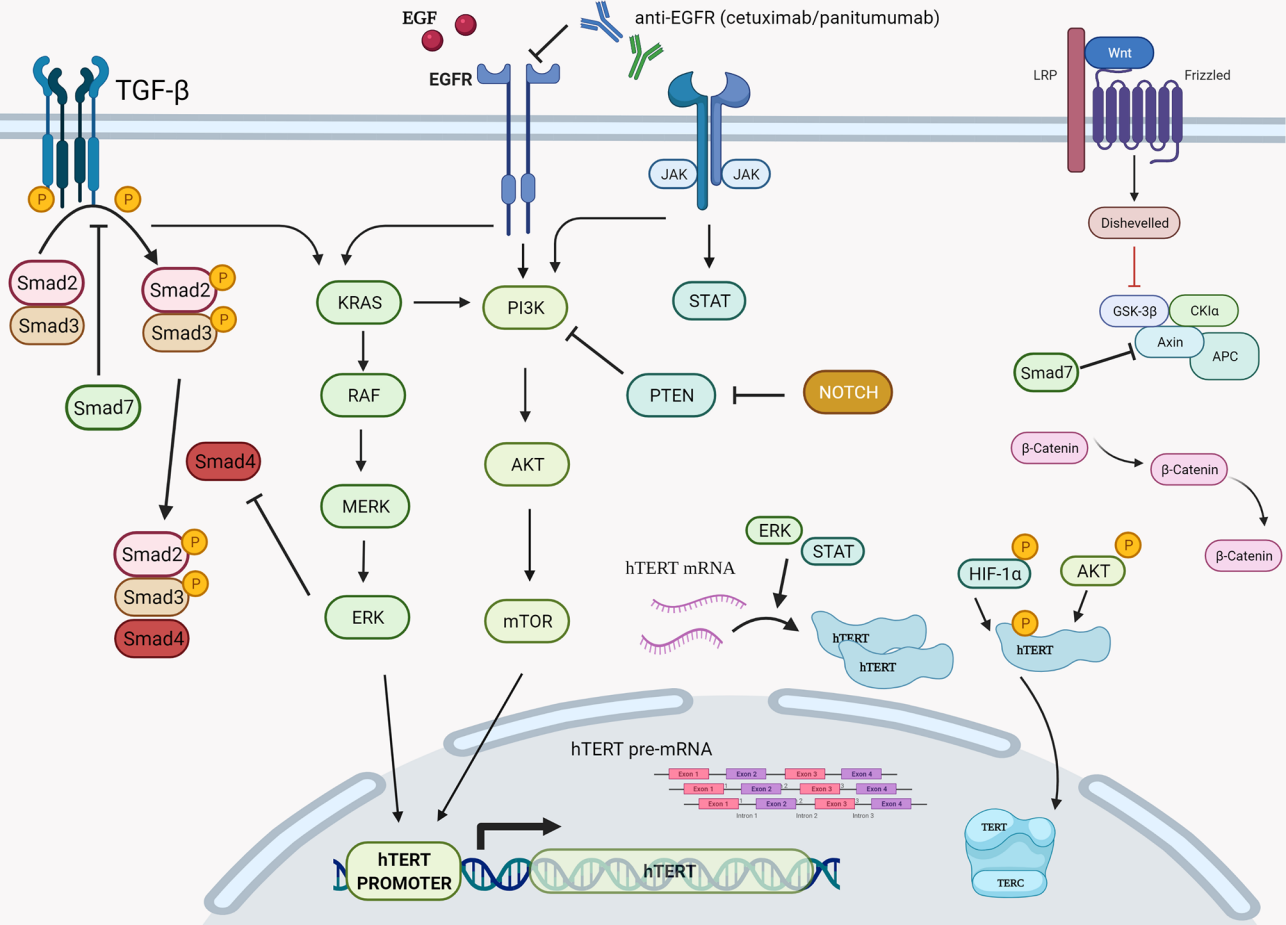
Graphical presentation of the various cellular cascades implicated in hTERT expression, telomerase activity, and telomere length. During transcription of hTERT gene, positive regulation is exerted *via* the PI3K/AKT/mTOR, the RAS/RAF/MEK/ERK 1/2, and the JAK/STAT pathways primarily on hTERT’s promoter. Another step of positive regulation is found during the translation and post translation phase of hTERT mRNA since the RAS/RAF/MEK/ERK pathway is described to regulate hTERT mRNA translation, while the PI3K/AKT/mTOR pathway seems to be involved post-translationally *via* direct phosphorylation of hTERT by AKT and HIF-1*α*. RAS, rat sarcoma; RAF, rapidly accelerated fibrosarcoma; MEK also known as MAP2K, MAPKK, mitogen-activated protein kinase kinase; ERK, also known as MAPK, extracellular signal-regulated kinase; PI3K, phosphatidylinositol 3-kinase; PKB, also known as Akt, protein kinase B; Mtor, mechanistic target of rapamycin kinase; HIF-1*α*, hypoxia-inducible factor-1*α*. Created with BioRender.com.

Driven by the need for effective prognostic and predictive biomarkers in CRC, our study focused on unveiling possible prognostic values of two novel biomarkers (MNf and TA) in PBLs isolated from patients with mCRC and laRC blood samples. Although our findings are hypothesis driven, given the relatively small number of patients we tested, our data provides further proof that MNf is not only significantly increased in CRC [as we have already shown (4)] but it could also serve as a promising biomonitor for mCRC and laRC prognosis. More specifically, the present study suggests that a decrease of MNf less than 29% between middle and initial MNf measurements can discriminate between progressive disease from stable/responsive disease with sensitivity of 36% and specificity of 87.0%, while if the threshold is set at 31% reduction of MNf then specificity reaches its highest value (87.2%). On the other hand, if the threshold of decrease is set at 29% for discrimination between stable/progressive and responsive disease then sensitivity reaches 72.7% and specificity 59.3%. **Figure 3** illustrates how MNf may follow a steep decreasing trend in patients with partial/complete response or a shallow decrease in patients with disease progression. These findings are important because early identification of those patients who are more likely to develop progressive disease can allow clinicians to take early decisions on therapy selection. A possible explanation of our findings lies in the fact that MN assay is a sensitive indicator of genomic damages of exogenous and endogenous origin (31, 32), while MNf in PBLs, despite the unknown underlying mechanism, is shown to positively correlate with chromosomal and genomic instability (5), which is one of the pillars in colorectal carcinogenesis. Therefore, the emergence of a resistant cluster of cancer cells against the sensitive “background” can be indirectly identified through MNf. However, as we have already shown (4), and according to our data from this study (not presented here), MNf can be increased after three months of treatment despite favorable disease response in case they are treated with combined treatment of FOLFIRI plus any biologic agent and then present a significant decrease at the end of the treatment. Therefore, the use of MNf as a prognostic biomarker in such patients may not be appropriate, and special caution is suggested for its use. Nonetheless, it could be used as a biomonitoring tool of cancer load. As for TA, even though the number of patients to which TA was evaluated is rather small, our results indicate its potential in CRC prognosis suggesting the need for future studies with greater patient sets. Despite the fact that statistical analysis between each response group did not reach statistical significance, our data indicate that TA is relatively higher in patients with progressive disease than those with partial or complete response at all sampling points. Moreover, in patients with progressive disease, an increase of TA is observed in the middle of their therapy, suggesting that patients who are more likely to develop progressive disease are more likely to have upregulated TA at the middle of their therapy. This can be explained by numerous studies that have identified telomerase as a key target of multiple carcinogenic pathways such as the PI3K/AKT/mTOR, the RAS/RAF/MEK/ERK 1/2, the JAK/STAT, and the JAK/PI3K/AKT/HSP90/mTORC1 (33–38). An interesting finding highlighting the complexity of hTERT regulation is that EGFR-mediated MAPK signaling attenuates Groucho-mediated gene repression, establishing a node for crosstalk between the EGFR, Notch, WNT, and TGF-*β* signaling pathways (39). A graphical presentation of the aforementioned mechanisms is shown in **Figure 4**. Unfortunately, since our primary objective was to investigate possible prognostic significance of these biomarkers, we did not examine any de-regulation of the aforementioned pathways.

Finally, in order to thoroughly understand and interpret our results, it is important to know how these biomarkers are affected by the different therapeutic agents used in our study. To begin with, FOLFOX, FOLFIRI, and FOLFOXIRI regimens are the three principal first-line therapeutic regimens administered for stage IV CRC according to HeSMO guidelines (20). They share two components, folinic acid (FA) and 5-fluorouracil (5-FU), while oxaliplatin and irinotecan are the compounds that differentiate the first two respectively or if combined make the later. CAPOX on the other hand, which along with FOLFOX is primarily used to treat laCRC (21), contains capecitabine and oxaliplatin. Based on bibliographic evidence validating the effect of these agents upon MNf and TA, it is evident that there is no universal effect. Overall, it is reported that 5-FU, capecitabine, oxaliplatin, and irinotecan increase BNMN (40–42), while FA significantly reduces it (43). Interestingly, as mentioned earlier, our data provides further proof that irinotecan and therefore FOLFIRI may increase MNf. However, for the most part, little data exists regarding any alterations of TA concomitant to administration of these substances. According to Akiyama et al., TA was decreased in human hematopoietic cancer cell lines, Daudi and U937, treated with irinotecan (44). However, there is no data suggesting any possible alterations of TA in CRC cell lines. Chung et al., using the HCT116 and DLD1 CRC cell lines, demonstrated a decreased TA concurrent to 5-FU administration possibly *via* STAT3 inhibition (a potent activator of hTERT promoter) (45). However, to the best of our knowledge, to date there is no study available evaluating the effect of the chemotherapeutic regimens of FOLFOX, FOLFIRI, FOLFOXIRI, or CAPOX on any of our biomarkers in this study. Given the great number of our patients treated with some kind of biologic agent (bevacizumab, cetuximab, panitumumab, or aflibercept), we expanded our research to include them as well. The only published data focusing on MNf comes from an *in vivo* cytogenetic assay performed in male Wistar rats, where cetuximab did not elicit any genotoxic effects (46). However, the results should be considered of limited value due to the lack of immunoreactivity of Cetuximab with rat tissues. To conclude, despite critical advances in most aspects of CRC management, it is indisputably one of the most important burdens of global health due to the related increased morbidity and mortality. Metastatic CRC, the final stage of CRC, remains a true challenge, not only for researchers and clinicians, but also for the socioeconomic system. This is because its inherent biologic complexity and diversity make it difficult to implement a universal approach in designing effective therapeutic and study protocols. On the contrary, even though laRC is a rather favorable type of CRC due to the absence of distant metastases, there still is a metastatic potential. Therefore, early recognition of chemoresistance is crucial. Our study made it possible not only to recognize possible prognostic significance at an early stage of therapeutic management for two novel biomarkers (MNf and TA), but also to suggest a relative threshold for MNf as a discrimination point between progressive and stable/responsive disease. However, the results of the current study should be interpreted with caution due to the limitations of the protocol (relatively small number of cases, different systemic treatment, different mutations subtypes, not randomized manner *etc.*) and could mainly serve as hypothesis generating study for further evaluation.

## Data Availability Statement

The raw data supporting the conclusions of this article will be made available by the authors without undue reservation.

## Ethics Statement

The studies involving human participants were reviewed and approved by the Human Ethics Committee at the University Hospital of Heraklion. The patients/participants provided their written informed consent to participate in this study.

## Author Contributions

TN conducted the experiments, interpreted the data, performed the analysis, and wrote the manuscript. EV conducted experiments. PS performed the analysis and interpreted the data. AA performed the statistical analysis, interpreted the data, and wrote the manuscript. AB conducted experiments. NR conducted experiments. JS provided blood samples, raised funds, edited, and critically revised the manuscript. AT raised funds, edited and critically revised the manuscript. JT conceived and designed the study, raised funds, edited, and critically revised the manuscript. All authors contributed to the article and approved the submitted version.

## Funding

This study was funded by Gastrointestinal Cancer Study Group (GIC-SG), Hellenic Society Medical Oncology (HESMO) and ToxPlus S.A. The funder, ToxPlus S.A., was not involved in the study design, collection, analysis, interpretation of data, the writing of this article or the decision to submit it for publication.

## Conflict of Interest

The authors declare that the research was conducted in the absence of any commercial or financial relationships that could be construed as a potential conflict of interest.
